# Severe Parvovirus B19–Associated Myocarditis in Children in the Post–COVID-19 Era: A Multicenter Observational Cohort Study

**DOI:** 10.1093/ofid/ofaf224

**Published:** 2025-04-11

**Authors:** Neal Russell, James Hatcher, Tim Best, Judith Breuer, James Charlesworth, Peter Muir, Barry Vipond, Stephane Paulus, Rohit Saxena, Jacob Simmonds, Stefania Vergnano, Peter Davis, Seilesh Kadambari

**Affiliations:** Department of Paediatric Infectious Diseases, Great Ormond Street Hospital for Children NHS Foundation Trust, London, UK; Department of Microbiology, Great Ormond Street Hospital for Children NHS Foundation Trust, London, UK; Infection, Immunity and Inflammation Department, University College London, Great Ormond Street Institute of Child Health, London, UK; Department of Microbiology, Great Ormond Street Hospital for Children NHS Foundation Trust, London, UK; Department of Microbiology, Great Ormond Street Hospital for Children NHS Foundation Trust, London, UK; Infection, Immunity and Inflammation Department, University College London, Great Ormond Street Institute of Child Health, London, UK; Department of Paediatrics, Oxford, UK; South West Regional Laboratory, UK Health Security Agency, Bristol, UK; South West Regional Laboratory, UK Health Security Agency, Bristol, UK; Department of Paediatrics, Oxford, UK; Cardiorespiratory and Critical Care Division, Great Ormond Street Hospital for Children NHS Foundation Trust, London, UK; Department of Paediatric Cardiology, Great Ormond Street Hospital for Children, London, UK; Paediatric Infectious Diseases and Immunology, University Hospitals Bristol and Weston NHS Foundation Trust, Bristol, UK; Paediatric Intensive Care Unit, Bristol Royal Hospital for Children, University Hospitals Bristol and Weston NHS Foundation Trust, Bristol, UK; Department of Paediatric Infectious Diseases, Great Ormond Street Hospital for Children NHS Foundation Trust, London, UK; Infection, Immunity and Inflammation Department, University College London, Great Ormond Street Institute of Child Health, London, UK

**Keywords:** epidemiology, myocarditis, parvovirus B19, pediatric, surveillance

## Abstract

This study describes a cluster of severe parvovirus B19–associated myocarditis cases in children across England in the context of an increase in circulating virus. Cases were identified across 3 large children's centers. Eight cases presented from 1 January 2019 to 31 December 2023 as compared with 19 from 1 January 2024 to 31 August 2024. Almost all (n = 25, 93%) required intensive care, and 24 (88%) received inotropes and 4 (15%) extracorporeal membrane oxygenation. Myocarditis appears to be temporally associated and a late sequela of parvovirus B19, resulting in high rates of intensive care unit admission. Testing with serology and blood polymerase chain reaction should be part of a syndromic screen for all children with severe myocarditis.

## Teaser

In the post COVID-19 era, there have been alerts across Europe of increases in circulating Parvovirus B19 infection. We present a cluster of paediatric patients in England, with severe Parvovirus associated myocarditis, requiring admission to the intensive care unit. Testing for Parvovirus, using serology and blood PCR, should be done as part of a syndromic screen in children with severe myocarditis.

Parvovirus B19 has been identified as a cause of myocarditis and dilated cardiomyopathy [1]. In June 2024, the European Centre for Disease Prevention and Control and the United Kingdom Health Security Agency released alerts of increases in circulating parvovirus B19 [2, 3]. We describe a cluster of severe myocarditis cases associated with parvovirus B19 infection across 3 large pediatric centers in England. We present diagnostic and epidemiologic evidence and compare the clinical severity in cases presenting before, during, and after the COVID-19 pandemic.

## METHODS

This retrospective observational cohort study was undertaken at Great Ormond Street Hospital for Children (London, England), Bristol Royal Hospital for Children (Bristol, England), and John Radcliffe Hospital (Oxford, England) from 1 January 2019 to 31 August 2024. All positive parvovirus test results were identified via local laboratory information management systems (positive IgM or blood polymerase chain reaction [PCR]), and myocarditis cases were identified from hospital discharge coding data (per *ICD-10*) in children aged <18 years. Hospital admissions related to parvovirus-associated myocarditis were defined as a positive virologic test result during admission and disease consistent with myocarditis based on clinical, laboratory, and cardiac assessment. This study received approval as a service evaluation (No. 3899) by the clinical audit/quality service at Great Ormond Street Hospital for Children, in line with the criteria outlined by the National Health Service's Health Research Authority and adhering to the ethical guidelines.

## RESULTS

**Figure 1.**
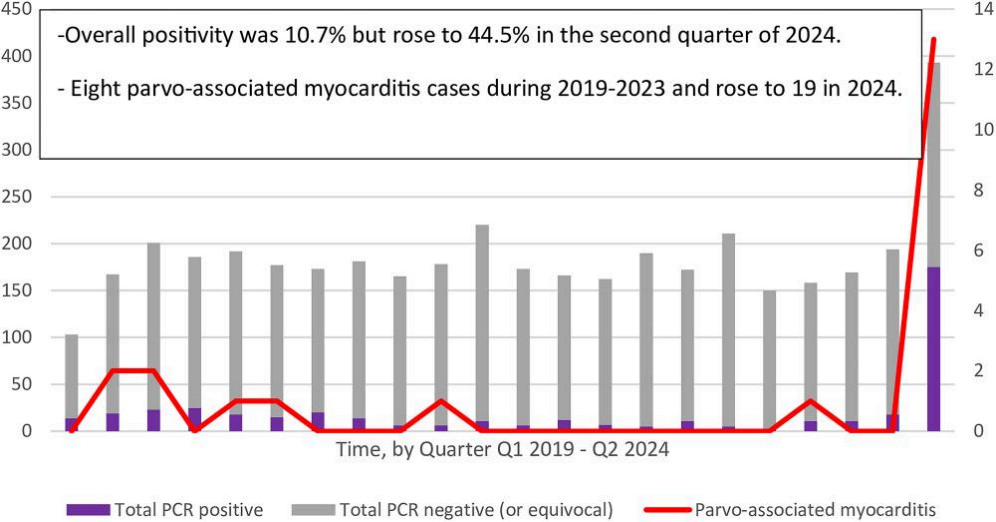
Parvovirus B19 testing, positivity rates, and associated myocarditis by quarter. Abbreviation: PCR, polymerase chain reaction.

During the period of study, we identified 27 children with parvovirus B19–associated myocarditis: 4 in 2019, 4 from 2020 to 2023, and 19 from 1 January 2024 to 31 August 2024. A total of 4081 parvovirus PCR tests were performed. The overall positivity was 10.7% (435/4081) but rose to 44.5% (175/393) in the second quarter of 2024 (Figure 1).

The median age was 21 months (IQR, 4 months–14 years), and 15 (55%) were female. The most frequent clinical symptoms were difficulty breathing (22/27, 81%), fatigue (15/27, 56%), and reduced feeding (8/27, 30%). Symptoms associated with acute parvovirus infection, such as fever (6/27, 22%) and rash (3/27, 11%), were uncommon (Table 1). Almost all cases (25/27, 93%) demonstrated severely impaired systolic function on echocardiogram (or cardiac magnetic resonance imaging, n = 1), among whom median ejection fraction at presentation was 25%. All children were in sinus rhythm except for 1 child who developed complete heart block, requiring insertion of a pacemaker. Successfully resuscitated cardiac arrest occurred in 3 children during the course of their intensive care admission.

**Table 1.** Clinical and Laboratory Presentation, Management, and Outcome of Parvovirus B19–Associated Myocarditis

	Age Group, y		
	0–1	1–4	5–18
No. of children	5	15	7
Sex: female	5 (100)	7 (47)	3 (43)
Parvovirus B19 PCR positive	5 (100)	15 (100)	7 (100)
Cycle threshold value, median	29.04	28.57	28.94
Parvovirus serology			
IgM positive	0	2	3
IgG positive	2	11	6
Presenting symptom			
Difficulty breathing	5 (100)	13 (87)	4 (57)
Cough/coryza		5 (33)	
Fatigue	2 (40)	9 (60)	4 (57)
Palpitations			1 (14)
Chest pain		1 (7)	2 (29)
Vomiting	2 (40)	5 (33)	3 (43)
Diarrhea	2 (40)		
Abdominal pain/distension			4 (57)
Dizziness/loss of consciousness		2 (15)	
Fever	1 (20)	5 (33)	
Rash			3 (43)
Presenting sign			
Tachypnea	5 (100)	9 (60)	4 (57)
Hypoxia		6 (40)	1 (14)
Cyanosis		4 (27)	
Tachycardia	4 (80)	9 (60)	4 (57)
Hepatomegaly	3 (60)	7 (47)	1 (14)
Cardiac marker			
Troponin	4/4 (100)	14/14 (100)	6 (86)
BNP elevated	5 (100)	15 (100)	6 (86)
CK >100		9/12 (75)	2/2 (100)
Echocardiography/cardiac MRI findings			
Impaired left ventricular function^a^	5 (100)	14 (93)	5 (71)
Moderate-severe mitral regurgitation	3 (60)	6 (33)	2 (29)
Left ventricular thrombus			1 (14)
Left ventricular ejection fraction, median, %	27	25	21
Electrocardiographic findings			
Sinus rhythm	2/3 (67)	11/11 (100)	4 (57)
Complete heart block			1 (14)
Ectopics			1 (14)
Nonspecific ST changes	2/3 (67)		2 (29)
T-wave inversion/flattening		8/11 (73)	
Prolonged QT		2/11 (18)	
Other complication			
Cardiopulmonary resuscitation	1 (20)		2 (29)
Left ventricular thrombus		1	1
Renal impairment		4	
Multiorgan failure		1	
Cerebral artery stroke			1
Red cell aplasia	1 (20)		

Data are presented as No. (%) unless noted otherwise. Blank cells indicate values of zero.

Abbreviations: BNP, brain natriuretic peptide; CK, creatine kinase; MRI, magnetic resonance imaging; PCR, polymerase chain reaction.

^a^Including biventricular and global.

All cases were parvovirus positive on blood PCR, with a median cycle threshold of 28.5 (minimum, 36.1; maximum, 11.5; 1 value was unquantifiable). Where quantitative PCR was performed, viral load in blood ranged from 17 700 to 708 000 IU/mL. Twenty (77%) children had serologic results available (IgG and IgM); among these, 19 were IgG positive but only 5 (25%) were IgM positive. One child had a positive PCR result for parvovirus B19 on endomyocardial biopsy. In terms of coinfection, nasopharyngeal airway testing revealed 5 cases of rhinovirus/enterovirus (undifferentiated), 2 Bocavirus (1 acquired after admission), 2 *Mycoplasma pneumoniae*, 1 *Chlamydia pneumoniae*, 1 coronavirus NL63, and 1 parainfluenza (type 3). In blood, 2 children were enterovirus positive (untypeable due to low viral load), 4 adenovirus (2 acquired after admission), 1 Epstein-Barr virus, and 1 human herpesvirus 6. Children were also assessed for noninfective causes of myocarditis according to local guidelines, and 1 child had muscular dystrophy and a second an atrial septal defect, which may have exacerbated the disease course.

Almost all (25/27, 93%) required admission to intensive care (median duration, 12 days), with 89% (24/27) receiving inotropes (median duration, 16 days) and 70% (19/27) invasive ventilation (median duration, 9.5 days). In addition, 15% (4/27) required extracorporeal membrane oxygenation, and 1 child required hemofiltration. Three children were treated with intravenous immunoglobulin, and all had severe disease: 1 required extracorporeal membrane oxygenation, and the other 2 had prolonged inotropes (median, 15.5 days) and intensive care admission (median, 36.5 days).

Overall, 7 of 8 (88%) of those admitted before 2024 survived to discharge; 14 of those admitted in 2024 were discharged with ongoing follow-up; and the remaining 5 were still inpatients at the time of writing. Among survivors diagnosed pre-2024, all have completed active management with apparent full recovery, while those diagnosed in 2024 all continue to improve but require ongoing medical management, such as aspirin, diuretics, angiotensin-converting enzyme inhibitors, β-blockers, and ivabradine, with 2 requiring low molecular weight heparin postdischarge for left ventricular thrombi.

Parvovirus B19–associated myocarditis presentations in 2024 were similar in age (median, 21 months vs 27 months pre-2024), length of intensive care admission (13 days in 2024 vs 11 days pre-2024), and cycle threshold values (median, 28.04 in 2024 vs 28.89 pre-2024) as compared with cases managed before and during the COVID-19 pandemic.

## DISCUSSION

We observed a significant cluster of severe parvovirus B19–associated myocarditis in children during 2024. This occurred in the context of much higher circulation of parvovirus B19 across Europe, as seen in the post–COVID-19 era during 2024, with reported increases in other severe parvovirus B19–related diseases such as fetal hydrops [4]. All 27 cases had parvovirus B19 DNA detected in blood samples, most at levels consistent with recent infection. However, only 25% of cases had detectable IgM antibody, suggesting that myocarditis cases may present relatively late in the course of infection or be due to a high number of false-negative findings on serology testing. This is in contrast to an outbreak of parvovirus B19–associated myocarditis in Italy during 2024, in which 75% (24/32) of affected cases had positive IgM antibody and parvovirus PCR results [5]. The higher rate of positive IgM results in the Italian study could be due to differences in the assay used or to obtaining samples earlier in the disease course. It is difficult to make any definitive serologic interpretation owing to the relatively small number of cases in both studies, and our findings emphasize the importance of parvovirus PCR testing in blood.

The current upsurge in parvovirus B19–associated myocarditis cases is consistent with increases in severe disease secondary to other viral infections, such as enteroviral meningitis and myocarditis, and respiratory syncytial virus disease affecting young children in the post–COVID-19 era [6–9]. The reasons for the increases are likely multifactorial and could be due to factors involving the host (ie, lack of exposure to common infections during the COVID-19 era leading to a susceptible population), pathogen (ie, increases in viral infections since societal restrictions lifted and changes in virulence factors), and society (ie, reduced access to primary care services leading to higher rates of severe disease presenting to secondary/tertiary care). There are few data comparing parvovirus B19–associated myocarditis with other viral etiologies in children. However, in children with viral myocarditis at 12-month follow-up from a large registry study, complete recovery was estimated to be 77%, comparable to our findings [10].

To our knowledge, we present the largest clinical and temporal cluster of parvovirus B19–associated myocarditis cases described in the literature. The temporal association with increased parvovirus circulation provides further epidemiologic evidence for the role of parvovirus infection in acute pediatric myocarditis. We found no significant differences in demography, clinical burden, or viral characteristics in affected children before, during, or after the COVID-19 pandemic. Moreover, the cases presented in this series are comparable to previously published series in age, severity, and clinical presentation [11–13].

Children had severe ventricular dysfunction on the background of structurally normal hearts and without underlying comorbidities in the majority of cases. Although most children survived and none in this cohort were listed for a cardiac transplant, there was frequent need for prolonged inotropic support and mechanical ventilation, with occasional extracorporeal membrane oxygenation and ongoing need for medical therapy and follow-up. Our clinical experience should emphasize the importance of parvovirus testing with blood PCR and serology, as part of a syndromic panel in all children with suspected myocarditis. Our experience also does not support the routine use of intravenous immunoglobulin in parvovirus B19–associated myocarditis, as outcomes in treated vs untreated children appeared similar.

This study has several limitations. Viral DNA detection in blood at the time of myocarditis diagnosis, although suggestive, does not prove causality. Myocardial detection with PCR could have better demonstrated causality, but a biopsy is rarely performed given the inherently high risk of the procedure. We did not use a specific case definition (ie, potentially overrelied on identifying cases on clinical discretion) or consistent screening tests (ie, the choice and type of infection screening tests done are varied across each center). The etiologic investigations for myocarditis and parvovirus infection across centers were not consistent and in some cases were incomplete, and the threshold for testing likely evolved during the study period. Some patients were also found to have other infections that may cause or contribute to myocarditis. Unfortunately, it was not possible to compare severity with nonparvovirus cases of myocarditis due to limitations in coding less severe cases in the earlier years of the study. Our 3 centers have large intensive care units with different referral patterns and may not be representative of the wider population, thereby leading to selection bias owing to the nature of the intensive care and cardiology services offered.

Larger studies should capture all causes of myocarditis cases, to see if there have been increases in other associated pathogens, and optimize testing protocols in suspected cases. Population-based surveillance of parvovirus infections relies on passive reporting from clinical centers. In the post–COVID-19 era, the resurgence of seemingly benign infections causing severe disease should encourage the development of active enhanced surveillance networks via sentinel sites to better capture current trends in circulating infections. This could enable earlier identification of outbreaks, which will inform public health measures and clinical practice.

## Note

***Potential conflicts of interest.*** All authors: No reported conflicts.
